# Objective differential diagnosis of Noonan and Williams–Beuren syndromes in diverse populations using quantitative facial phenotyping

**DOI:** 10.1002/mgg3.1636

**Published:** 2021-03-27

**Authors:** Antonio R. Porras, Marshal Summar, Marius George Linguraru

**Affiliations:** ^1^ Sheikh Zayed Institute for Pediatric Surgical Innovation Children’s National Hospital Washington D.C. USA; ^2^ Department of Biostatistics and Informatics Colorado School of Public Health University of Colorado Anschutz Medical Campus Aurora CO USA; ^3^ Rare Disease Institute – Genetics and Metabolism Children’s National Hospital Washington D.C. USA; ^4^ School of Medicine and Health sciences George Washington University Washington D.C. USA

**Keywords:** facial analysis, facial phenotyping, machine learning, Noonan, Williams–Beuren

## Abstract

**Introduction:**

Patients with Noonan and Williams–Beuren syndrome present similar facial phenotypes modulated by their ethnic background. Although distinctive facial features have been reported, studies show a variable incidence of those characteristics in populations with diverse ancestry. Hence, a differential diagnosis based on reported facial features can be challenging. Although accurate diagnoses are possible with genetic testing, they are not available in developing and remote regions.

**Methods:**

We used a facial analysis technology to identify the most discriminative facial metrics between 286 patients with Noonan and 161 with Williams‐Beuren syndrome with diverse ethnic background. We quantified the most discriminative metrics, and their ranges both globally and in different ethnic groups. We also created population‐based appearance images that are useful not only as clinical references but also for training purposes. Finally, we trained both global and ethnic‐specific machine learning models with previous metrics to distinguish between patients with Noonan and Williams–Beuren syndromes.

**Results:**

We obtained a classification accuracy of 85.68% in the global population evaluated using cross‐validation, which improved to 90.38% when we adapted the facial metrics to the ethnicity of the patients (*p* = 0.024).

**Conclusion:**

Our facial analysis provided for the first time quantitative reference facial metrics for the differential diagnosis Noonan and Williams–Beuren syndromes in diverse populations.

## Graphical abstract

Although distinctive facial features have been reported between Noonan and Williams–Beuren syndromes, studies have found a variable incidence of those characteristics in populations with diverse ancestry. We sought to identify and quantify facial features discriminative of these two syndromes in population with diverse ancestry using facial analysis. We provide for the first time quantitative reference facial metrics in diverse populations that can be used both at the clinic and for training purposes.

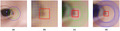

## 1 INTRODUCTION

Noonan syndrome is a congenital genetic disorder that affects between 1 per 1000 and 1 per 2500 live births (Noonan, 1994; Nora, 1974), and it is caused by different mutations in several genes (OMIM #163950, #605275, #609942, #610733, #611553, #613224, #613706, #615355, #616559, #616564, #618499, #618624 or #619087). Subjects with Noonan syndrome typically present characteristic facial features and short stature (Allanson et al., 2010; van der Burgt et al., 1999), and about half have congenital cardiac abnormalities (Noonan, 1994). Although it is generally diagnosed based on the observation of key features, molecular testing can provide a confirmation of diagnosis in about 70% of the cases (Allanson & Roberts, 1993; Bhambhani et al., 2014). An early diagnosis is not only important for a prompt treatment but also to provide genetic counseling to the family. However, early diagnosis of Noonan syndrome is challenging and late diagnoses are frequent, with reports showing an average age of diagnosis of 9 years (Sharland et al., 1992).

The differential diagnosis of Noonan syndrome includes Williams–Beuren syndrome (OMIM #194050) (Allanson, 1987; Morris, 1993), among other disorders. Williams–Beuren syndrome has a prevalence of about 1 in 7500 live births (Strømme et al., 2002)^,^ and patients with this condition present similar characteristics to patients with Noonan syndrome, including facial dysmorphology and short stature (Allanson, 1987; Cassidy & Allanson, 2010; Morris, 1993). Williams–Beuren syndrome is also associated with congenital heart disease (Morris, 1993, 2010). As both the physical manifestations and their severity are variable, individuals with Williams–Beuren syndrome are often undetected during early childhood, with an average diagnostic age of 3.66 years (Huang et al., 2002). Diagnostic confirmation of Williams–Beuren syndrome is often attained using fluorescence in situ hybridization, but it can also be established using other techniques such as array comparative genomic hybridization (Pober, 2010).

Diagnostic tests are typically requested after the identification of signs and symptoms associated with either Noonan or Williams–Beuren syndrome, and they are often not available in developing countries. In many cases, the examination is made based only on phenotypical observations and symptoms, which may lead to errors and delays in the correct diagnosis. Although several studies have reported independently similar facial phenotypes among patients with Noonan and Williams–Beuren syndrome, there are also studies reporting distinctive facial features specific to each syndrome (Allanson, 1987; Castelo‐Branco et al., 2007; Digilio & Marino, 2001; Morris & Mervis, 2000; Noonan, 1994; Romano et al., 2010; Winter et al., 2018; Wu et al., 1999). However, even though these distinctive observations are often found in patients presenting either Noonan or Williams–Beuren syndromes, they are not always present and they are modulated by the ethnic background of the patients(Kruszka, Porras, Addissie, et al., 2017; Kruszka et al., 2018). An objective and accurate way to differentiate between these two genetic syndromes can significantly improve the clinical management of these patients and their outcomes.

In this work, we use a digital facial analysis technology to objectively quantify and illustrate facial phenotypical differences between patients with Noonan and Williams–Beuren syndrome. We use our technology to determine a set of objective metrics that can be used as a reference to help differentiating between these two syndromes. As the phenotype of genetic syndromes is modulated by the ethnic background of the patients (Kruszka, Addissie, et al., 2017; Kruszka, Porras, Addissie, et al., 2017; Kruszka et al., 2018; Kruszka, Porras, Sobering, et al., 2017), we also present the metrics that are relevant for patient populations from four different ethnic groups: African descent, Asian, Caucasian, and Latin American.

### 1.1 State of the art

The phenotypical observations of patients with Williams–Beuren and Noonan syndromes have been studied independently in the literature (Allanson, 1987, 2016; Kruszka, Porras, Addissie, et al., 2017; Kruszka et al., 2018; Morris, 1993, 2010; Noonan, 1994; Roberts et al., 2013). Some studies have reported similar facial observations among patients with either of those syndromes: hypertelorism (Allanson, 1987; Levin & Enzenauer, 2017; Noonan, 1994; Wu et al., 1999), telecanthus (Castelo‐Branco et al., 2007; Chen, 2012; Morris & Mervis, 2000; Romano et al., 2010), ptosis (Allanson, 2016; Digilio & Marino, 2001; Winter et al., 2018), epicanthal folds (Allanson, 2016; Kruszka et al., 2018; Morris, 1993; Roberts et al., 2013), and short nose (Allanson, 2016; Kruszka et al., 2018; Morris, 1993; Roberts et al., 2013). However, other studies have reported distinctive facial features between patients with Williams–Beuren and Noonan syndromes. Patients with Noonan syndrome are often described as presenting low‐set ears and widely spaced eyes (Bertola et al., 2006; Essawi et al., 2013; Kruszka, Porras, Addissie, et al., 2017; Rokhaya et al., 2014; Şimşek‐Kiper et al., 2013), whereas patients with Williams–Beuren syndrome are described as presenting a short nose and a wide mouth (Kruszka et al., 2018; Patil et al., 2012; Pérez Jurado et al., 1996). Other discriminative facial features reported include down‐slanted palpebral fissures in patients with Noonan syndrome (Bertola et al., 2006; Essawi et al., 2013; Hung et al., 2007; Kruszka, Porras, Addissie, et al., 2017; Şimşek‐Kiper et al., 2013) and a long philtrum in patients with Williams–Beuren syndrome (Kruszka et al., 2018; Patil et al., 2012; Pérez Jurado et al., 1996). However, as given in Table 1, variable reports on the incidence of these observations suggest that those characteristics are not discriminative for an accurate differential diagnosis based on physical observations between Noonan and Williams–Beuren syndromes. Only 17% of the patients with Noonan syndrome from Senegal study (Rokhaya et al., 2014) and 58% of the patients from Turkey study (Şimşek‐Kiper et al., 2013) were reported as presenting low‐set ears. When patients with Noonan syndrome were stratified based on the ethnic background (Kruszka, Porras, Addissie, et al., 2017), 82% of African descent, 94% of Asian, and 88% of Latin American patients presented low‐set ears. Similarly, the incidence reports of widely spaced eyes in patients with Noonan syndrome ranged from the 44% reported (Bertola et al., 2006) in a Brazilian population to the 100% reported (Rokhaya et al., 2014) for a patient population from Senegal, and (Hung et al., 2007) for a population from Taiwan.

**TABLE 1:** Reported incidence of discriminative facial features between patients with Noonan and Williams–Beuren syndromes in different studies and populations

Noonan syndrome					
Study	Population	Low ears	Down‐slanted eyes	Widely spaced eyes	Epicanthal folds
Rokhaya et al. (2014)	Senegal	17%	Not reported	100%	Not reported
Şimşek‐Kiper et al. (2013)	Turkey	58%	73%	85%	Not reported
Essawi et al. (2013)	Egypt	57%	100%	100%	Not reported
Hung et al. (2007)	Taiwan	Not reported	59%	Not reported	56%
Bertola et al. (2006)	Brazil	Not reported	66%	44%	Not reported
Yoshida et al. (2004)	Japan	Not reported	Not reported	100%	Not reported
Kruszka, Porras, Addissie, et al. (2017)	African	82%	87%	80%	70%
	Asian	94%	86%	96%	64%
	Latin American	88%	73%	94%	55%

Williams–Beuren syndrome					
Study	Population	Wide mouth	Short nose	Long philtrum	Epicanthal folds
Patil et al. (2012)	India	100%	100%	85%	52%
Pérez Jurado et al. (1996)	Mixed	Not reported	90%	83%	71%
Kruszka et al. (2018)	African	88%	88%	88%	13%
	Asian	78%	75%	79%	63%
	Latin American	91%	74%	93%	73%

On the other hand, only 78% of the Asian population with Williams–Beuren syndrome (Kruszka et al., 2018) presented a wide mouth, as compared to the 100% reported (Patil et al., 2012) for an Indian population. When looking at the nose size, 100% of patients from India presented a short nose (Patil et al., 2012), compared with 74% of Latin American (Kruszka et al., 2018).

To the best of our knowledge, quantitative methods to distinguish between patients with Noonan and Williams–Beuren syndrome have been explored only in the study by Preus (Preus, 2008). In that study, a clustering analysis showed that patients with Noonan and Williams–Beuren syndrome are clinically distinguishable. However, that study focused on many clinical observations that are not easily observable. For instance, cardiac abnormalities cannot be observed without the specialized equipment, which may not be available in in rural areas and developing countries. Similarly, although family history information is essential for an early diagnosis, it is sometimes unknown to the clinical team. In addition, that previous study analyzed a small population of patients, it did not provide objective metrics that can be translated into direct clinical use, and it did not consider the ethnic variability of the patients.

In the current study, we provide reference facial metrics adapted to the ethnic background of the patients that can be used directly at any clinic. In addition, we illustrate facial appearance features that can be quantified by computer methods, but only qualitatively assessed by the human eye, and which are relevant to differentiate between Noonan and Williams–Beuren syndrome. To the best of our knowledge, this is the first time that facial analysis technology is used to quantify and illustrate graphically on population‐based computer‐generated images the specific facial features that allow for the distinction of these two genetic syndromes in diverse populations, in addition to providing reference geometric measurements.

## 2 METHODS

### 2.1 Data

We evaluated the face photographs of 286 (49 infants, 47 toddlers, 71 children, 28 adolescents, and 91 adults; 150 male and 136 female) individuals with Williams–Beuren syndrome from 19 countries, and 161 (45 infants, 29 toddlers, 47 children, 18 adolescents, and 22 adults; 93 male and 68 female) patients with Noonan syndrome from 14 countries. All participants were diagnosed with molecular testing and/or clinical evaluation by local expert geneticists. Verbal or written formal consent from the parent/guardian was obtained by local institutional review boards and the protocol #7134 at the Children's National Hospital. A subset of these dataset is publicly available through the “Atlas of Human Malformation Syndromes in Diverse Populations” of the National Human Genome Research Institute – National Institutes of Health (Muenke et al., 2016). Clinical findings and additional details on these data can be found in previous studies (Kruszka, Porras, Addissie, et al., 2017; Kruszka et al., 2018). We categorized the patients into four groups: African descent (28 patients with Williams–Beuren and 35 with Noonan syndrome), Asian (26 patients with Williams–Beuren and 40 with Noonan syndrome), Caucasian (121 patients with Williams–Beuren and 40 with Noonan syndrome), or Latin American (111 patients with Williams–Beuren and 46 with Noonan syndrome). In this study, we only included those patients whose face photographs were frontal, with eyes open, and with even illumination conditions. We discarded all pictures with illumination artifacts or shadows that could affect the appearance of the face. We also discarded pictures in which any part of the face was not totally visible (e.g., glasses, hair over the eyes).

### 2.2 Facial analysis

The facial analysis methods used in this study are based on the technology previously described (Cerrolaza et al., 2016; Ojala et al., 1996). We have used that technology to identify Down (Kruszka, Porras, Sobering, et al., 2017), 22q11.2 deletion (Kruszka, Addissie, et al., 2017), Noonan (Kruszka, Porras, Addissie, et al., 2017), and Williams–Beuren syndromes (Kruszka et al., 2018) from healthy individuals in diverse populations.

#### 2.2.1 Quantification of facial features

**FIGURE 1:**
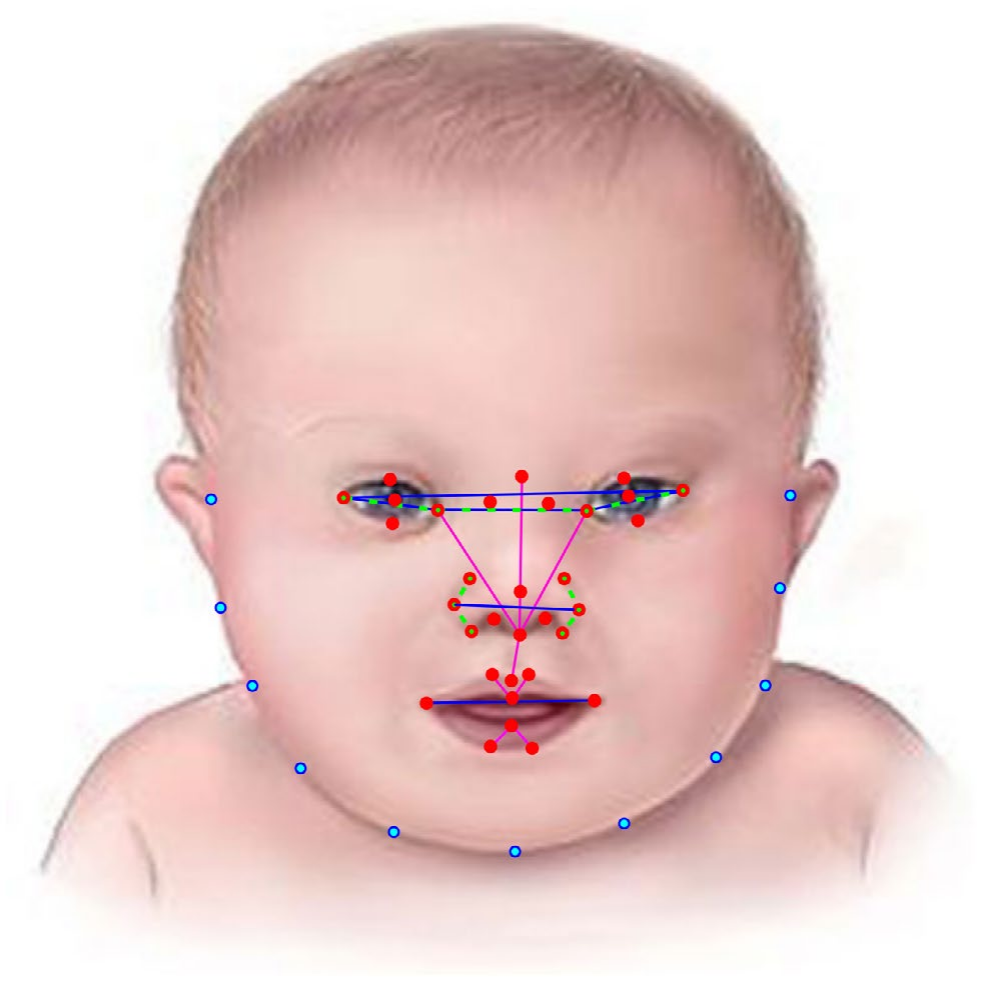
Representation of the facial landmarks and geometric metrics. Inner facial landmarks are represented as red circles. Horizontal distances between these landmarks are represented as blue lines. Vertical distances are represented as magenta lines. Angles are represented with green dashed lines, with the center of the angle represented as a green circle around the landmark, and the extremes represented with a green dot inside the landmark

**FIGURE 2:**
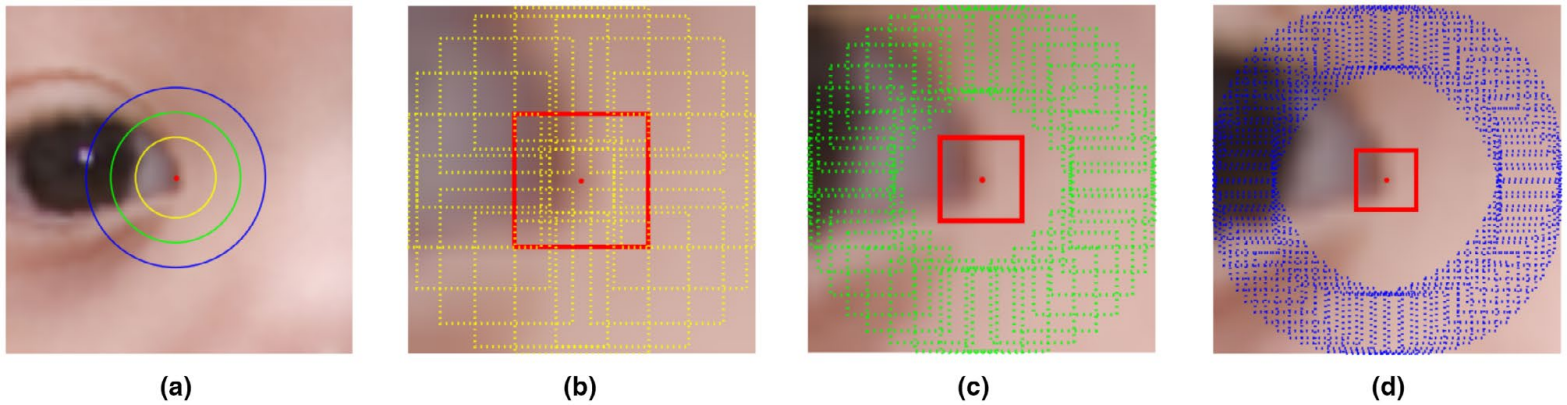
Representation of the image patches used to calculate the local binary patterns (LBP) around the medial canthi of the right eye. (a) the area around the landmark that is involved in the calculation of the LBPs at the three resolutions, in yellow for the highest resolution (R1), green for a medium resolution (R2), and blue for the lowest resolution (R3). (b), (c), and (d) illustrate the image patches involved in the calculation of the LBP at resolution levels R1, R2, and R3, respectively. At each level, the LBPs are calculated by comparing the image patch around the landmark (in red) with the patches in their neighborhood (in yellow for R1, green for R2, and blue for R3)

Our face analysis technology quantifies a set of geometric measurements (i.e., distances and angles) from 44 anatomical facial landmarks (e.g., lateral canthi, oral commissures…). The location of each of the landmarks and the geometric measurements is represented in Figure 1. We estimated the average of the measurements on the right and left sides of the face to obtain symmetric metrics that are easier to interpret and to use as clinical references, and their absolute differences to quantify asymmetry. All horizontal measurements were normalized with respect to the ear‐to‐ear distance, and all vertical measurements were normalized to the distance between the mid‐point between the oral commissures and the nose root. Asymmetry measurements were normalized with respect to the average value from the measurements at the left and right sides. In addition, our technology quantifies the appearance around each of a subset of 33 inner facial landmarks using texture descriptors based on local binary patterns (LBP) as represented in Figure 2 (Cerrolaza et al., 2016; Ye et al., 2005), which are sensitive to lines, shadows, and local intensity contrast.

#### 2.2.2 Feature selection and classification

Once all geometric and appearance metrics were calculated, we selected the most discriminative ones between Noonan and Williams–Beuren syndrome using recursive feature elimination (Guyon et al., 2002) based on a support vector machine (SVM) classifier (Cortes & Vapnik, 1995). To compensate for the different number of patients with Noonan and Williams–Beuren syndromes, we used a weighting scheme (Du & Chen, 2005) that balanced the contribution of each individual to the SVM classifier, therefore the total weight of the patients with Noonan and Williams–Beuren syndrome was the same. We evaluated our classifier using leave‐one‐out cross‐validation (Devijver & Kittler, 1982) for increasing numbers of features, and we selected the optimal as the minimum number of features at which the area of the receiving operator characteristic curve converged (Bradley, 1997). In addition to the optimal list of features obtained, we also estimated the individual discriminative power of each feature using the non‐parametric Mann–Whitney U test (Mann & Whitney, 1947).

We performed the above process to obtain the optimal list of features that are discriminative in the global population, regardless of the ethnic background of the patients. Then, we repeated it for each different population, thus obtaining a list of optimal discriminant features adapted to the ethnicity of the patients. Finally, we compared the performance of the global and the ethnic‐specific models in discriminating between Williams–Beuren and Noonan syndromes.

## 3 RESULTS

We obtained an average accuracy of 85.68% in the discrimination of patients with Noonan syndrome and Williams–Beuren syndrome in the global population using the list of 14 optimal facial features identified by our face analysis technology. Specifically, we obtained accuracies of 87.58% and 84.62% in the correct identification of Noonan and Williams–Beuren syndrome, respectively. The list of optimal geometric and appearance features, their distribution, and individual p‐value in the global population can be consulted in our supplementary material. The clinical interpretation of those features is given in Table 2, organized according to the region of the face at which they were observed: eyes, nose, and mouth.

**TABLE 2:** Interpretation of the quantitative results in the global population

	Significant differences		Relevant differences	
	Noonan	Williams–Beuren	Noonan	Williams–Beuren
Eyes	More pronounced hypertelorism and telecanthus	More pronounced down‐slanted palpebral fissures	Higher orbital rim	Smaller palpebral fissures
Nose		Longer nasal alas Shorter nose	More asymmetric nasal bridge	
Mouth		Thicker lower lip Wider mouth		

We obtained average accuracies of 93.65%, 87.88%, 91.30%, and 89.17% in the African descent, Asian, Caucasian, and Latin American populations, respectively, when using population‐specific models. As with the global population, the details of the geometric and appearance facial features can be consulted in our supplementary material. Table 3 gives our interpretation of the optimal features identified for each population.

**TABLE 3:** Interpretation of the quantitative results in the African descent, Asian, Caucasian, and Latin American populations. Characteristics not observed in the global population are indicated in green

	Significant differences		Relevant differences	
	Noonan	Williams–Beuren	Noonan	Williams–Beuren
African descent population				
Eyes	More pronounced hypertelorism	Smaller palpebral fissures with more significant ptosis		Smaller palpebral fissures More asymmetric palpebral fissures
Nose				Thicker/more rounded nasal lobe More asymmetric nasal alas
Mouth		Thicker lower lip Wider mouth		
Asian population				
Eyes		More pronounced down‐slanted palpebral fissures		Smaller palpebral fissures More asymmetric palpebral fissures
Nose				Longer nasal alas
Mouth		Thicker lower lip Wider mouth		More asymmetric philtrum and cupid's bow
Caucasian population				
Eyes	More pronounced hypertelorism and telecanthus	More pronounced down‐slanted palpebral fissures	Higher orbital rim	More pronounced ptosis
Nose		More asymmetric nasal alas and lobe		Shorter nose
Mouth		Thicker lower lip More asymmetric upper lip thickness Wider mouth		
Latin American population				
Eyes	More pronounced hypertelorism Higher orbital rim			Smaller palpebral fissures
Nose		Shorter nose		
Mouth		Thicker lower lip Wider mouth		More asymmetric lips Flatter philtrum and cupid's bow

Table 4 gives the accuracy in differentiating between Noonan and Williams–Beuren syndromes of the models created both for the global population and for each population included in this study. Similar to our previous works identifying genetic syndromes from a healthy population(Cerrolaza et al., 2016; Kruszka, Addissie, et al., 2017; Kruszka, Porras, Addissie, et al., 2017; Kruszka et al., 2018; Kruszka, Porras, Sobering, et al., 2017; Zhao et al., 2014), we obtained improved results when we adapted our technology to specific ethnic groups. In average, we obtained an improvement of 5.49% when using specific models for each ethnicity, with a p‐value of 0.024 estimated using a Fisher's exact test. However, our results also show that the improvement is only statistically significant (*p* < 0.05) on the Caucasian population, with a p‐value of 0.044.

**TABLE 4:** Comparison of the accuracy obtained with the global model (trained with all ethnic groups) and with the specific model trained with a specific ethnic group on each population

Ethnicity	Global model	Ethnicity‐specific model	Improvement	*p*‐value*
African descent	87.30%	93.65%	7.27%	0.363	
Asian	84.85%	87.88%	3.57%	0.800	
Caucasian	83.23%	91.30%	9.70%	0.044	
Latin American	86.62%	89.17%	1.91%	0.727	
Global population	85.68%	90.38%	5.49%	0.024	

* *p*‐value calculated using a Fisher's exact test.

## 4 DISCUSSION

Despite many phenotypical similarities reported in the literature between patients with Noonan and Williams–Beuren syndrome (e.g., short stature, ptosis, down‐slanted palpebral fissures, cardiac abnormalities) (Allanson, 1987; Morris, 1993, 2010; Noonan, 1994; Roberts et al., 2013), our facial analysis demonstrated that these two genetic conditions can be distinguished in the global population with accuracy higher than 85% based only on facial observations. Patients with Noonan syndrome present significantly more pronounced hypertelorism and telecanthus, whereas patients with Williams–Beuren syndrome present significantly more down‐slanted palpebral fissures, shorter nose with longer alas, and a wider mouth with a thicker lower lip. In addition, patients with Noonan syndrome are likely to have higher orbital rim and a more asymmetric nasal bridge, and patients with Williams–Beuren syndrome often present smaller and less rounded palpebral fissures, although differences between the two populations in these observations were not found to be statistically significant when evaluated individually.

Our results also indicate that the physical manifestations are modulated by the ethnic background of the patients. Similar to previous works classifying individuals with genetic syndromes from healthy subjects (Kruszka, Addissie, et al., 2017; Kruszka, Porras, Addissie, et al., 2017; Kruszka et al., 2018; Kruszka, Porras, Sobering, et al., 2017), we obtained a higher classification accuracy when we adapted the list of relevant discriminative facial features to specific ethnic groups. Our results show that, although the features described above are discriminative between Noonan and Williams–Beuren syndromes in the global population, there are other features that can be more discriminant on specific populations, either individually or combined with previous features.

In the African‐descent population, unlike the global population, the palpebral slanting angle is not essential to discriminate Williams–Beuren and Noonan syndrome. Patients of this ethnic group with Williams–Beuren syndrome often present a more rounded nasal lobe and asymmetric nasal alas, and more asymmetric palpebral fissures. Importantly, although these features combined were relevant to identify patients with Williams–Beuren syndrome from Noonan syndrome, they were not found to be significantly different between the two populations when evaluated individually.

In the Asian population, a wider mouth with a thicker lower lip and more down‐slanted palpebral fissures were significant to distinguish patients with Williams–Beuren syndrome from patients with Noonan syndrome. Moreover, patients with Williams–Beuren syndrome often showed more asymmetry in the palpebral fissures and in the cupid's bow and philtrum, in addition to smaller palpebral fissures and longer nasal alas. Differences in these features were not statistically significant when compared individually with patients with Noonan syndrome.

We identified similar discriminative features in the Caucasian population that those found in the general population except for the nasal observations. Moreover, in this population, patients with Williams–Beuren syndrome presented significantly more asymmetric nasal alas and lobe than patients with Noonan syndrome, and a significantly more asymmetric upper lip. They often presented shorter nose as well, although differences with respect to patients with Noonan syndrome were not found to be statistically significant.

The Latin American population with Noonan syndrome showed a significantly higher orbital rim and more pronounced hypertelorism. Patients with Williams–Beuren syndrome presented a significantly wider mouth with a thicker lower lip, and a shorter nose. They often presented smaller palpebral fissures and a flatter philtrum and cupid's bow, but these features were not found to be significantly different between the two populations when evaluated individually.

Although ethnic‐specific classification models provided a higher accuracy compared with the model created from the global population, this improvement was statistically significant only for patients from the Caucasian population. One possible explanation for this is a lower phenotypical variability of the Caucasian population used in this work compared with the other ethnic groups. To categorize patients, we followed the racial and ethnic categories used by the National Institutes of Health. However, the Asian population analyzed in this work includes patients from China, India, and Malaysia, thus introducing a high ethnic variability in the Asian group. This higher variability makes it difficult to find ethnic‐specific features, which translate into a classification model with an accuracy that is higher in average but not significantly different to the model built from the global population. As more data become available, it will be possible to focus on the study of more specific populations.

Although many of the discriminant facial observations between Noonan and Williams–Beuren syndromes found are consistent among ethnicities (i.e., more significant hypertelorism in patients with Noonan syndrome and wider mouth in patients with Williams–Beuren syndrome), there are a few observations that are specific to each ethnic group and that can be subtle to the human eye. However, they can be quantified using a systematic analysis as presented in this work. Our facial analysis technology uses an objective and quantitative approach to identify and stratify facial phenotypes, which is essential to detect those subtle facial features that are indicators of genetic conditions. In this work, we used this technology not only to distinguish patients with Noonan and Williams–Beuren syndromes, but also to provide reference metrics that can be used in any clinic. Moreover, these metrics were objectively defined for different ethnic groups, which resulted in improved accuracy for the potential diagnosis of the syndromes from phenotypical observations. Our results show the potential of our facial analysis technology to support the assessment of patients with genetic syndromes in areas of the world with diverse populations and where access to specialists is sometimes limited.

Finally, we also used our technology to create population‐based computer‐generated images that illustrate the specific appearance of relevant facial features for the differential diagnosis of Noonan and Williams–Beuren syndromes. These images can be used as a reference for the identification of these syndromes in populations with different ethnic background, both for training and diagnostic purposes. However, other observations from clinical evaluation as well as family history or behavioral observations, if they are available, provide additional information that needs to be considered for a clinical diagnosis.

## CONFLICT OF INTEREST

The authors do not have any conflicts of interest that are relevant to this manuscript.

## AUTHOR CONTRIBUTIONS

All authors conceptualized this work together. A.R.P and M.G.L. designed the methods. A.R.P. implemented the methods, performed the experiments, and wrote the initial draft of the manuscript. M.G.L. reviewed the results and revised the manuscript. M.S. provided the clinical perspective and revised the results and manuscript.

## Supporting information

Supplementary Material

## Data Availability

A subset of the facial photographs used in this study are available through the “Atlas of Human Malformation Syndromes in Diverse Populations” of the National Human Genome Research Institute – National Institutes of Health (Muenke et al., 2016). The discriminative facial metrics between Noonan and Williams‐Beuren syndromes and their ranges in diverse population are available as supplementary material of this article.
