# Supplementary material for: Objective differential diagnosis of Noonan and Williams–Beuren syndromes in diverse populations using quantitative facial phenotyping
Source: Mol Genet Genomic Med. 2021 Mar 27;9(5):e1636. doi: 10.1002/mgg3.1636 (PMC8172204; doi:10.1002/mgg3.1636)
Supplement: Supplementary file 1 — Supplementary Material [file MGG3-9-e1636-s001.pdf]

Supplementary material

In this document, we present the results obtained using our facial analysis technology to distinguish patients with Williams-Beuren syndrome from patients with Noonan syndrome based on their facial phenotype, both in the global population and in populations with African-descent, Asian, Caucasian and Latin American ancestry. The location of each of the 44 facial landmarks analyzed and the geometric metrics calculated from them are represented in Figure 1. Local binary patterns (LBP) were used to quantify the image appearance around each facial landmark, as represented in Figure 2. They were calculated by comparing the image patch around that landmark (in red in Figure 2) and a set of image patches in its neighborhood at different resolutions (in yellow, green and blue in Figure 2).

For each patient group, we present the following information:

- Population size and classification results, including the optimal number of features selected, and the variation of the sensitivity, specificity, accuracy and area under the receiver operator characteristic (ROC) curve with respect to the number of features.
- The list of discriminant geometric features selected, their distribution in the patients with Williams-Beuren and Noonan syndromes, and their individual and independent p-values estimated using a non-parametric Mann-Whitney test.
    - (H) next to a feature indicates that it is a horizontal distance normalized with respect to the ear-to-ear distance.
    - (V) next to a feature indicates that it is a vertical distance normalized with respect to the distance between the nose root and the mid-point between the oral commissures.
    - Angles are measured in degrees.
    - Asymmetry metrics are normalized to the average value of the left and right sides.
- The list of discriminant appearance features selected, their individual and independent p-values estimated using a non-parametric Mann-Whitney test, and the population-based computer-generated images illustrating their differences between patients with Williams-Beuren and Noonan syndromes. We present two types of appearance features:
    - Average texture: the appearance image is the average between the left and right sides of the face.
    - Asymmetry of texture: the appearance image is the absolute difference between the left and right side of the face, in which brighter shades of gray represent higher values.

On each appearance image, we represent: 1) the landmark location and the image patch around it in red; 2) the center of each neighbor patch in blue, with which the patch around the landmark location is compared to calculate the LBP; 3) and the image area involved in the calculation of the LBPs delimited with two green circles. Moreover, we included some markers in red in the form of arrows and lines to help identifying differences of appearance between patients with Noonan and Williams-Beuren syndromes.

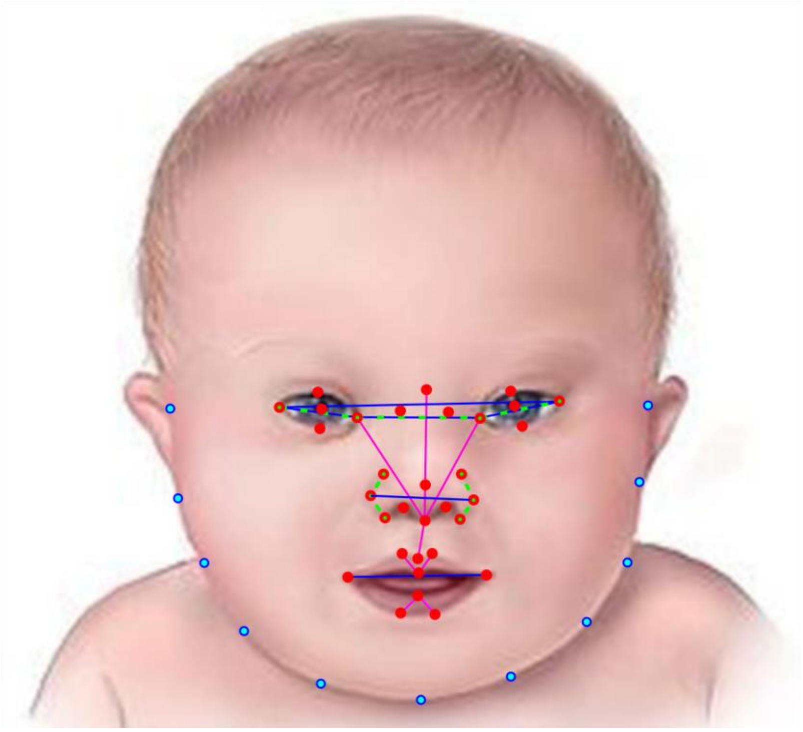

Figure 1. Representation of the facial landmarks and geometric metrics. Inner facial landmarks are represented as red circles. Horizontal distances between these landmarks are represented as blue lines. Vertical distances are represented as magenta lines. Angles are represented with green dashed lines, with the center of the angle represented as a green circle around the landmark, and the extremes represented with a green dot inside the landmark.

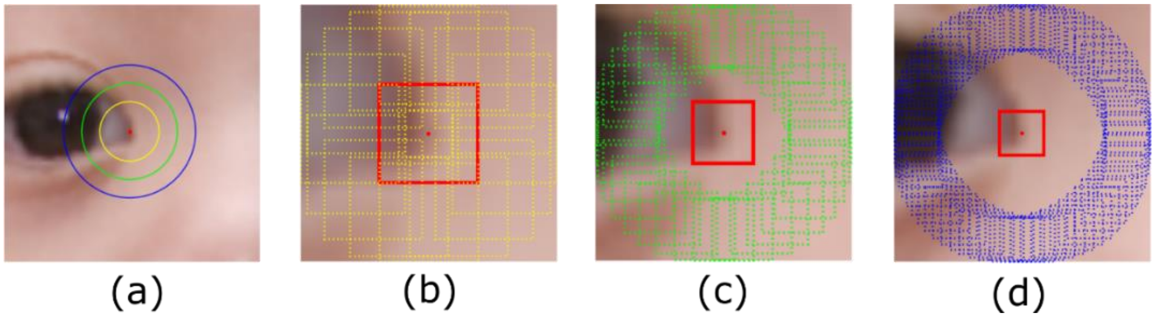

Figure 2. Representation of the image patches used to calculate the local binary patterns (LBP) around the medial canthi of the right eye. (a) shows the area around the landmark that is involved in the calculation of the LBPs at the three resolutions, in yellow for the highest resolution (R1), green for a medium resolution (R2), and blue for the lowest resolution (R3). (b), (c), and (d) illustrate the image patches involved in the calculation of the LBP at resolution levels R1, R2, and R3, respectively. At each level, the LBPs are calculated by comparing the image patch around the landmark (in red) with the patches in their neighborhood (in yellow for R1, green for R2, and blue for R3), as explained in [Cerrolaza et al.,"Identification of dysmorphic syndromes using landmark-specific local texture descriptors", IEEE International Symposium on Biomedical Imaging, pp.1080-1083, 2016].

Global population

Area under ROC curve: 92.51%.

Optimal number of features: 14.

	Patients	Accuracy (%)
**Noonan**	161	87.58
**Williams-Beuren**	286	84.62
**Global**	447	85.68

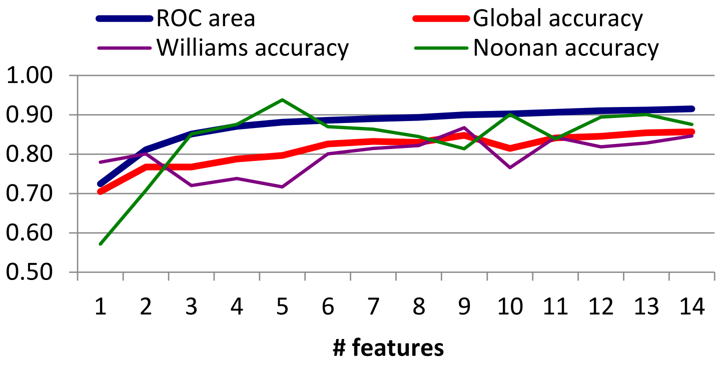

Geometric features	Noonan		Williams-Beuren		p-value
	Average	Std	Average	Std	
Distance between medial canthi (H)	0.294	0.027	0.275	0.023	<0.001
Lower lip thickness (V)	0.173	0.024	0.192	0.027	<0.001
Distance between oral commissures (H)	0.364	0.060	0.413	0.071	<0.001
Distance between lateral canthi (H)	0.681	0.049	0.652	0.037	<0.001
Palpebral slanting angle	-2.299	2.312	-3.227	2.751	<0.001
Nasal alas angle	103.098	4.237	101.695	6.751	<0.001
Nose length (V)	0.815	0.063	0.799	0.073	4.97E-02
Distance between columella and cupid's bow (V)	0.254	0.040	0.252	0.041	7.73E-01

Appearance features	Observations	p-value
Average texture at lateral of lower lip vermillion (R2)	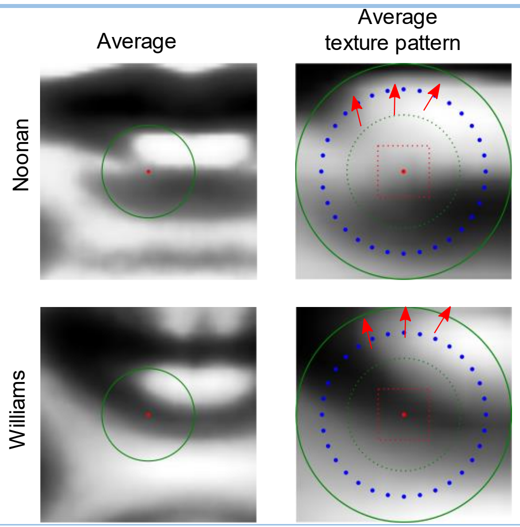	<0.001
Interpretation: Patients with Williams-Beuren syndrome have a thicker lower lip than patients with Noonan syndrome.		

Average texture at top of nasal alas (R2)	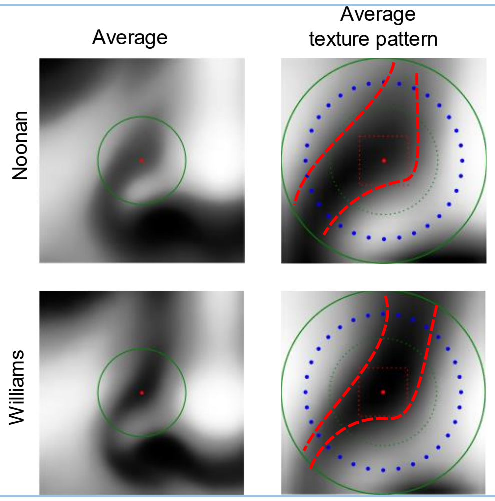	1.96E-02
Interpretation: Patients with Williams-Beuren syndrome present longer nasal alas than patients with Noonan syndrome.		
Average texture at lateral canthi (R2)	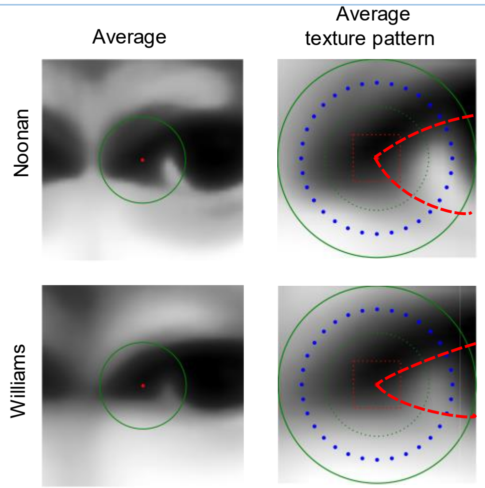	1.24E-01
Interpretation: Patients with Noonan syndrome have more rounded palpebral fissures and patients with Williams-Beuren syndrome present smaller palpebral fissures.		
Texture at nasal root (R1)	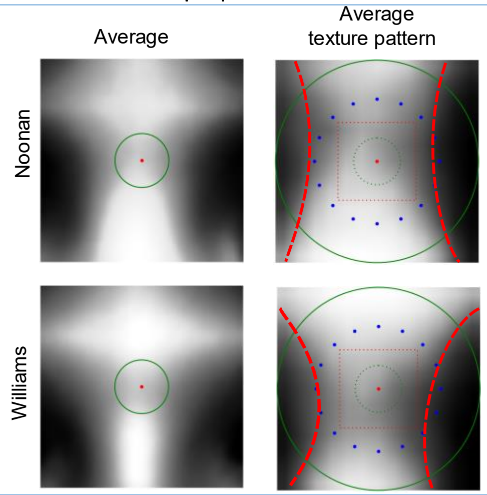	2.18E-01
Interpretation: Patients with Noonan syndrome present higher orbital rims than patients with Williams-Beuren syndrome.		

<table>
<tr><td>Asymmetry of texture at lateral of nasal bridge (R1)</td><td>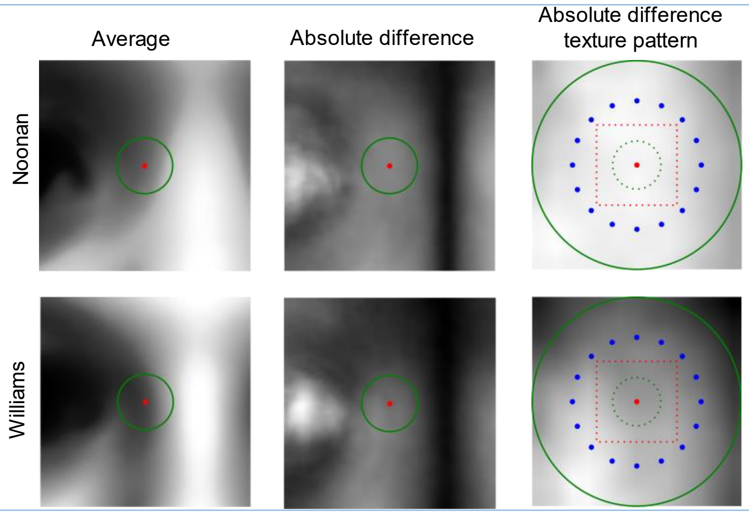
</td><td>2.95E-01</td></tr>
<tr><td colspan="3">Interpretation: Patients with Noonan syndrome present a more asymmetric nasal bridge than patients with Williams-Beuren syndrome.</td></tr>
<tr><td>Average texture at oral commissures (R1)</td><td>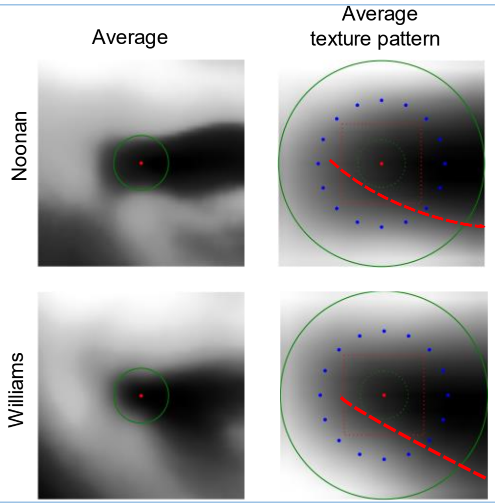
</td><td>5.29E-01</td></tr>
<tr><td colspan="3">Interpretation: Patients with Williams-Beuren syndrome have thicker lower lips around the oral commissures than patients with Noonan syndrome.</td></tr>
</table>

African descent population

Area under ROC curve: 96.12%.

Optimal number of features: 9.

	Patients	Accuracy (%)
**Noonan**	35	88.57
**Williams-Beuren**	29	100.00
**Global**	64	93.65

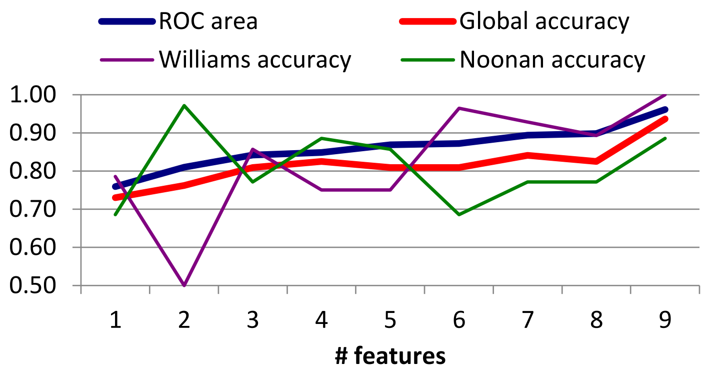

Geometric features	Noonan		Williams-Beuren		p-value
	Average	Std	Average	Std	
Lower lip thickness (V)	0.175	0.019	0.203	0.031	<0.001
Distance between medial canthi (H)	0.307	0.029	0.289	0.024	8.76E-03
Distance between oral commissures (H)	0.357	0.055	0.400	0.089	6.28E-02

Appearance features	Observations	p-value
Average texture at lateral canthi (R2)	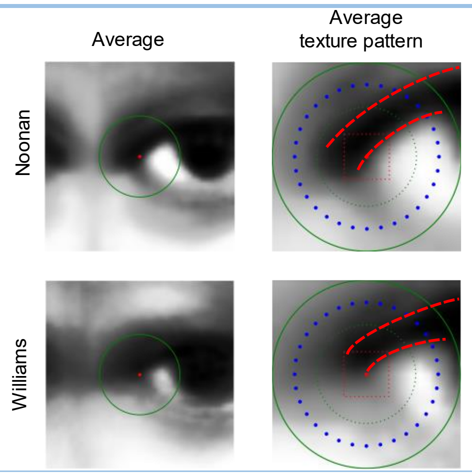	1.07E-03
Interpretation: patients with Noonan syndrome have bigger and more rounded palpebral fissures than patients with Williams-Beuren syndrome, who present more significant ptosis.		

<table>
<tr><td>Asymmetry of texture at lateral canthi (R2)</td><td>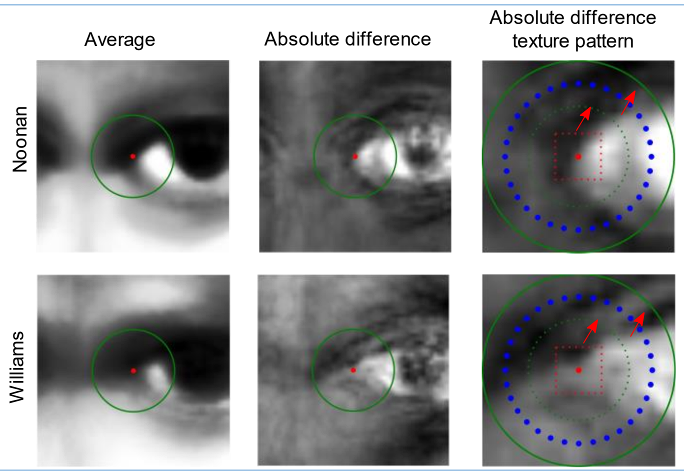
</td><td>2.32E-01</td></tr>
<tr><td colspan="3">Interpretation: patients with Williams-Beuren syndrome present more asymmetric palpebral fissures than patients with Noonan syndrome.</td></tr>
<tr><td>Texture at columella (R1)</td><td>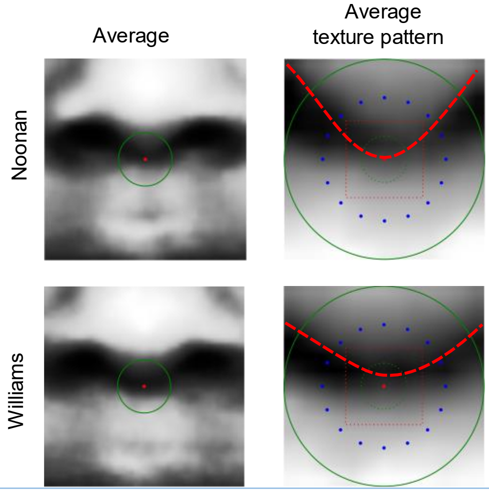
</td><td>2.48E-01</td></tr>
<tr><td colspan="3">Interpretation: patients with Williams-Beuren syndrome present a more rounded nasal lobe than patients with Noonan syndrome.</td></tr>
<tr><td>Average texture at upper eyelids (R1)</td><td>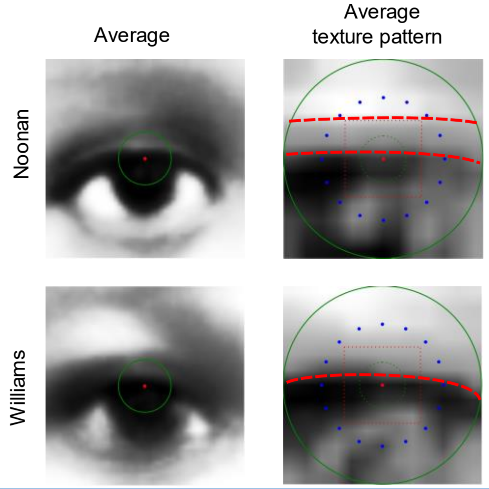
</td><td>2.90E-01</td></tr>
<tr><td colspan="3">Interpretation: patients with Williams-Beuren syndrome present more pronounced ptosis than patients with Noonan syndrome.</td></tr>
</table>

<table>
<tr><td>Asymmetry of texture at bottom of nasal alas (R3)</td><td>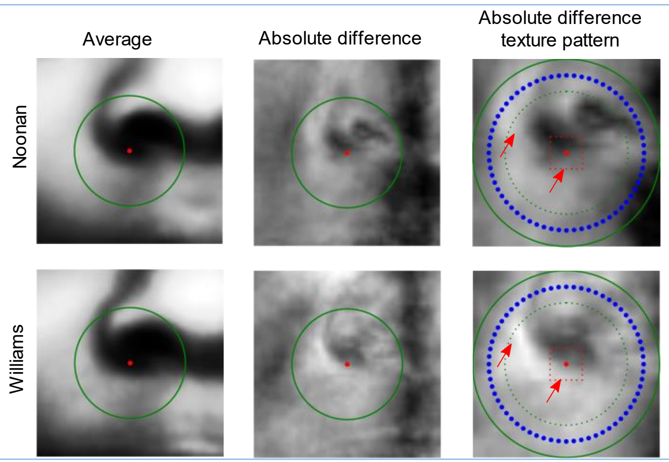
</td><td>6.23E-01</td></tr>
<tr><td colspan="3">Interpretation: Patients with Williams-Beuren syndrome present more asymmetric nasal alas than patients with Noonan syndrome.</td></tr>
<tr><td>Average texture at center of the eyes (R1)</td><td>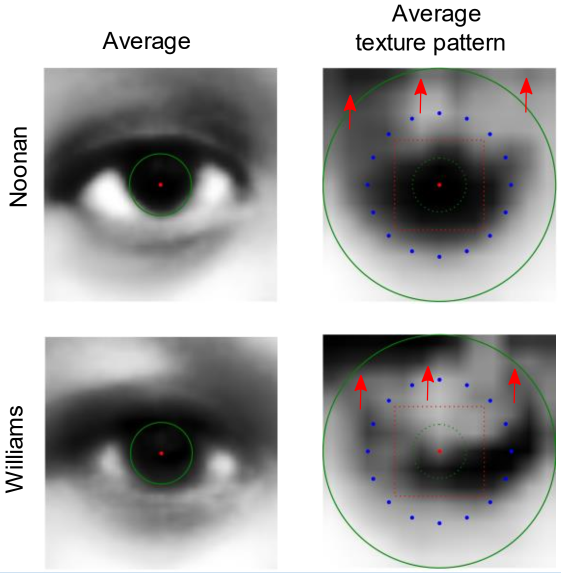
</td><td>9.17E-01</td></tr>
<tr><td colspan="3">Interpretation: patients with Williams-Beuren syndrome have smaller palpebral fissures than patients with Noonan syndrome.</td></tr>
</table>

Asian population

Area under ROC curve: 90.58%.

Optimal number of features: 10.

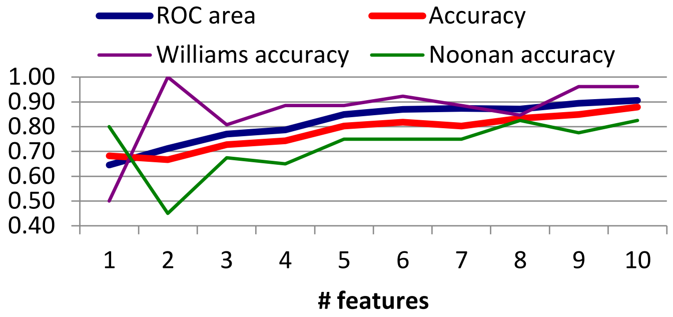

	Patients	Accuracy (%)
**Noonan**	40	82.50
**Williams-Beuren**	26	96.15
**Global**	66	87.88

Geometric features	Noonan		Williams-Beuren		p-value
	Average	Std	Average	Std	
Lower lip thickness (V)	0.170	0.021	0.184	0.020	2.38E-03
Distance between oral commissures (H)	0.339	0.054	0.384	0.071	8.51E-03
Palpebral slanting angle	-2.485	2.224	-3.774	1.972	2.53E-02
Asymmetry in distance between medial and lateral canthi (H)	0.007	0.006	0.011	0.009	1.11E-01

Appearance features	Observations	p-value
Average texture at center of the eyes (R2)	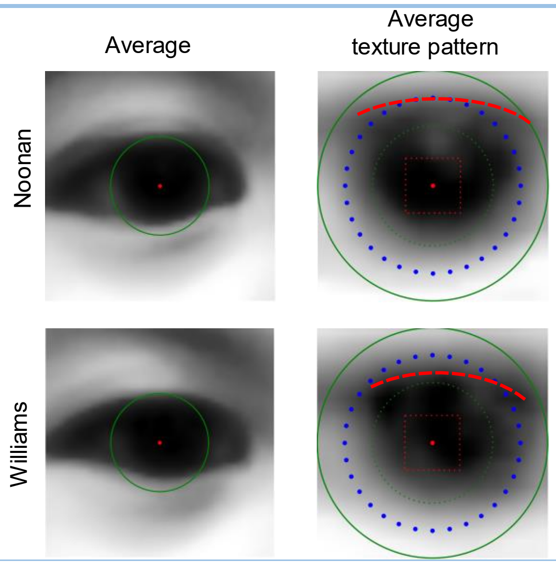	1.17E-01
Interpretation: patients with Williams-Beuren syndrome smaller palpebral fissures than patients with Noonan syndrome.		

Average texture at bottom of nasal alas (R2)	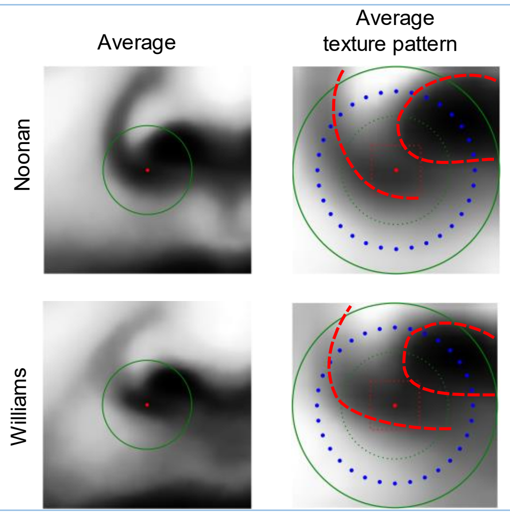	2.91E-01
Interpretation: Patients with Williams-Beuren syndrome have longer nasal alas that patients with Noonan syndrome, who present more rounded alar rims.		
Average texture at bottom of nasal alas (R3)	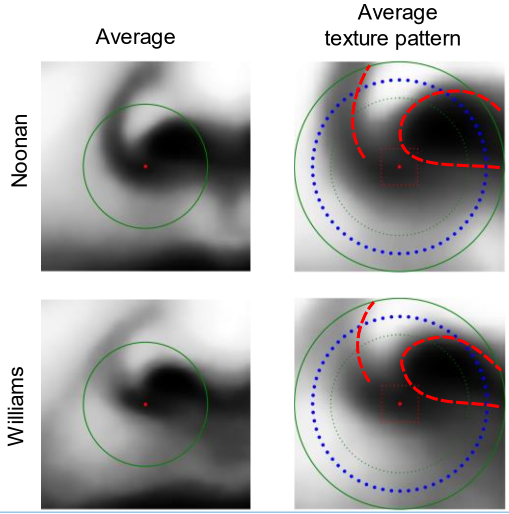	2.97E-01
Interpretation: Patients with Williams-Beuren syndrome have longer nasal alas than patients with Noonan syndrome, who present more rounded alar rims.		
Asymmetry of texture at lower eyelids (R3)	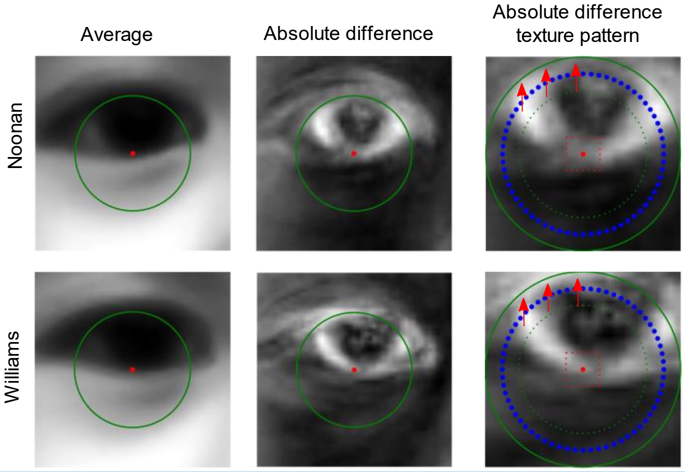	4.58E-01
Interpretation: Patients with Williams-Beuren syndrome have more asymmetric palpebral fissures than patients with Noonan syndrome.		

Asymmetry of texture at lateral of cupid's bow (R3)	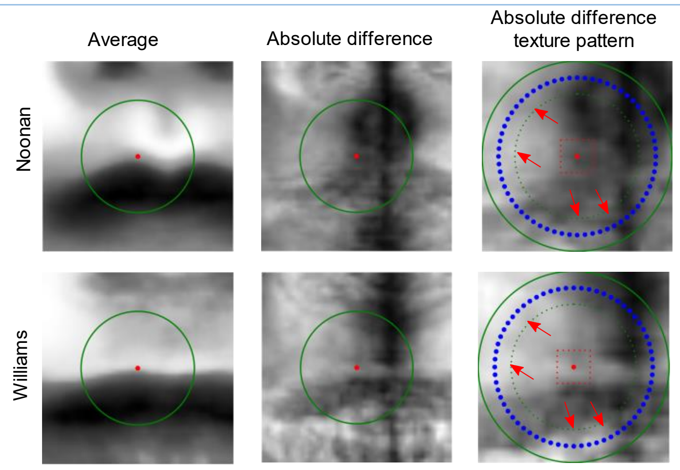	9.32E-01
Interpretation: Patients with Williams-Beuren syndrome present more asymmetric upper lip than patients with Noonan syndrome.		
Asymmetry of texture at center of the eyes (R1)	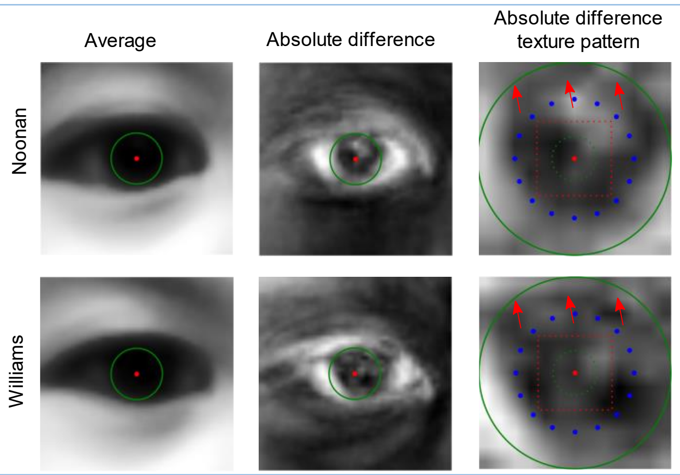	9.95E-01
Interpretation: Patients with Williams-Beuren syndrome present more asymmetric palpebral fissures than patients with Noonan syndrome.		

Caucasian population

Area under ROC curve: 95.08%.

Optimal number of features: 10.

	Patients	Accuracy (%)
**Noonan**	40	92.50
**Williams-Beuren**	121	90.91
**Global**	161	91.30

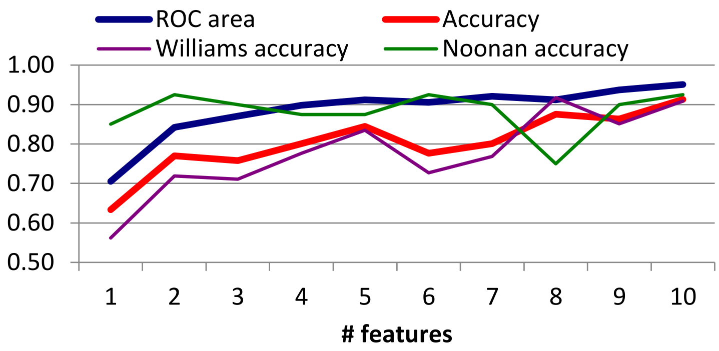

Geometric features	Noonan		Williams-Beuren		p-value
	Average	Std	Average	Std	
Distance between medial canthi (H)	0.289	0.017	0.272	0.024	<0.001
Lower lip thickness (V)	0.172	0.030	0.188	0.024	<0.001
Asymmetry in upper lip thickness (V)	0.008	0.006	0.015	0.011	<0.001
Distance between oral commissures (H)	0.373	0.054	0.421	0.067	<0.001
Distance between lateral canthi (H)	0.668	0.029	0.648	0.033	1.11E-03
Palpebral slanting angle	-1.202	2.380	-2.547	2.721	5.12E-03
Nose length (V)	0.817	0.057	0.798	0.081	1.65E-01

<table>
<tr><th>Appearance features</th><th>Observations</th><th>p-value</th></tr>
<tr><td>Asymmetry of texture at nostrils (R1)</td><td>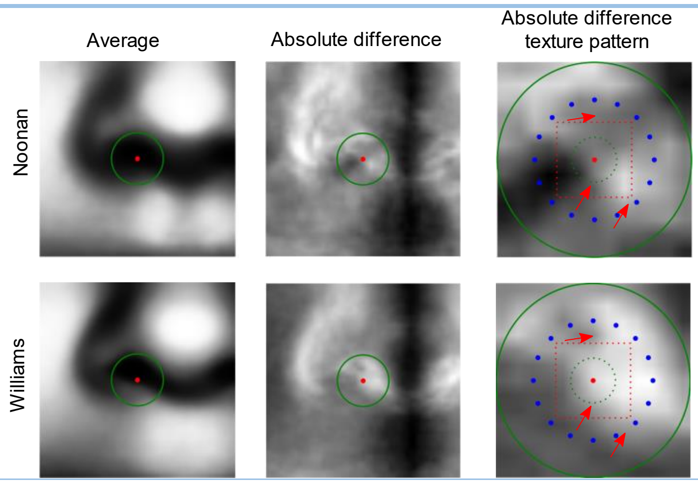
</td><td>2.98E-02</td></tr>
<tr><td colspan="3">Interpretation: Patients with Williams-Beuren syndrome present more asymmetric nasal alas and lobe than patients with Noonan syndrome.</td></tr>
</table>

Average texture at lateral canthi (R1)	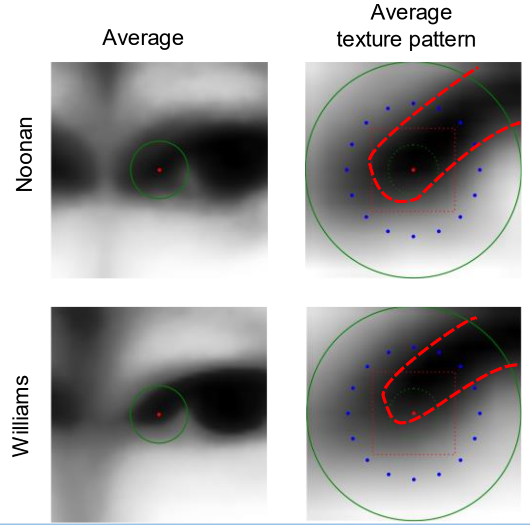	7.48E-02
Interpretation: Patients with Williams-Beuren syndrome present more pronounced ptosis than patients with Noonan syndrome.		
Texture at nasal root (R1)	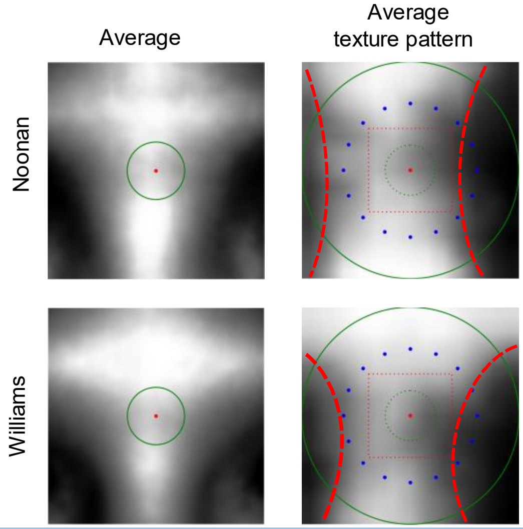	1.83E-01
Interpretation: Patients with Noonan syndrome present higher orbital rims than patients with Williams-Beuren syndrome.		

Latin American population

Area under ROC curve: 93.75%.

Optima number of features: 13.

	Patients	Accuracy (%)
**Noonan**	46	91.30
**Williams-Beuren**	111	88.28
**Global**	157	89.17

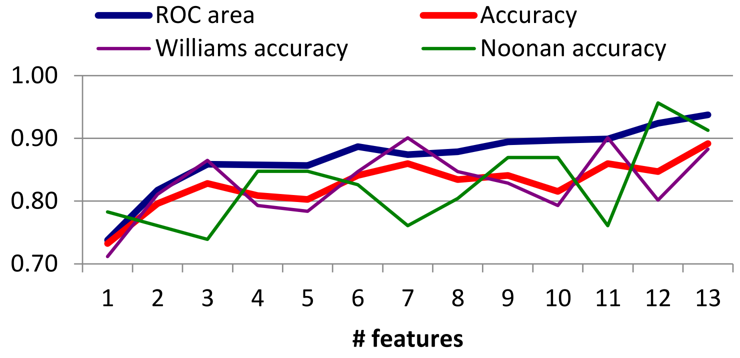

Geometric features	Noonan		Williams-Beuren		p-value
	Average	Std	Average	Std	
Lower lip thickness (V)	0.174	0.019	0.196	0.027	<0.001
Distance between medial canthi (H)	0.291	0.024	0.274	0.019	<0.001
Distance between oral commissures (H)	0.382	0.064	0.413	0.068	7.23E-03
Nose length (V)	0.836	0.068	0.805	0.065	1.52E-02
Asymmetry in lower lip thickness (V)	0.013	0.017	0.013	0.011	2.85E-01

Appearance features	Observations	p-value
Texture at nasal root (R3)	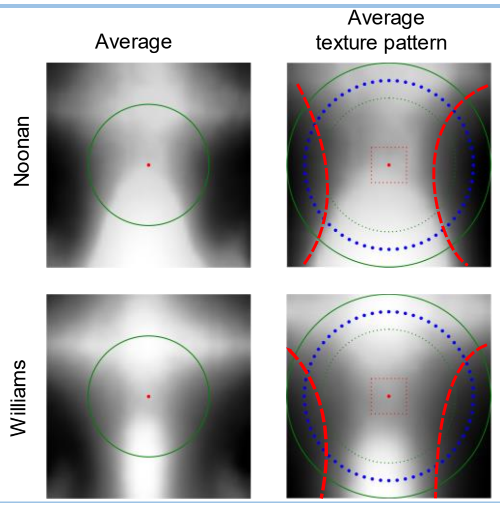	7.53E-05
Interpretation: Patients with Noonan syndrome present higher orbital rims than patients with Williams-Beuren syndrome.		

Average texture at center of the eyes (R3)	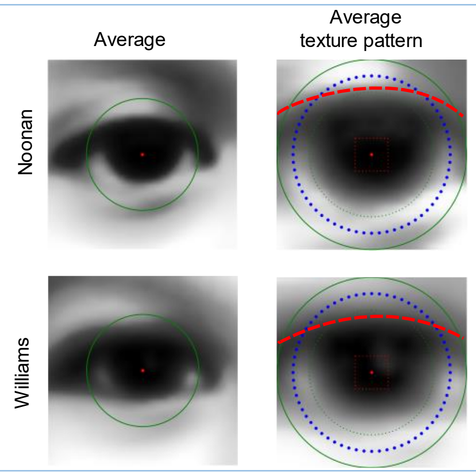	4.00E-02
Interpretation: Patients with Williams-Beuren syndrome present smaller palpebral fissures than patients with Noonan syndrome.		
Asymmetry of texture at oral commissures (R1)	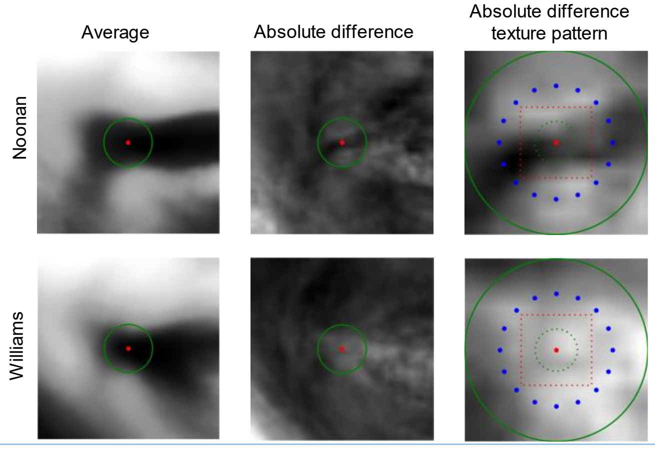	2.19E-01
Interpretation: Patients with Williams-Beuren syndrome present more asymmetric oral commissures than patients with Noonan syndrome.		
Texture at cupid's bow (R3)	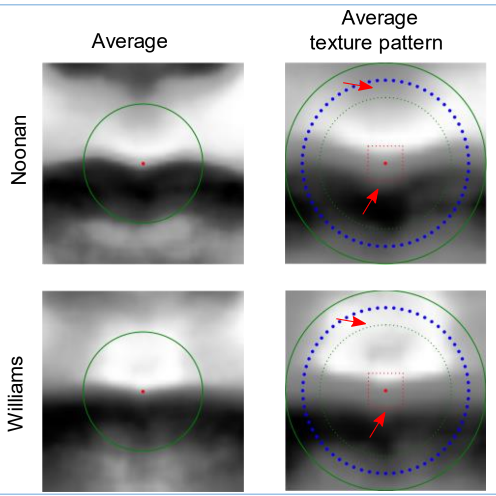	2.51E-01
Interpretation: Patients with Williams-Beuren syndrome present flatter cupid's bow and philtrum than patients with Noonan syndrome.		

<table>
<tr><td>Asymmetry of texture at top of nasal alas (R2)</td><td>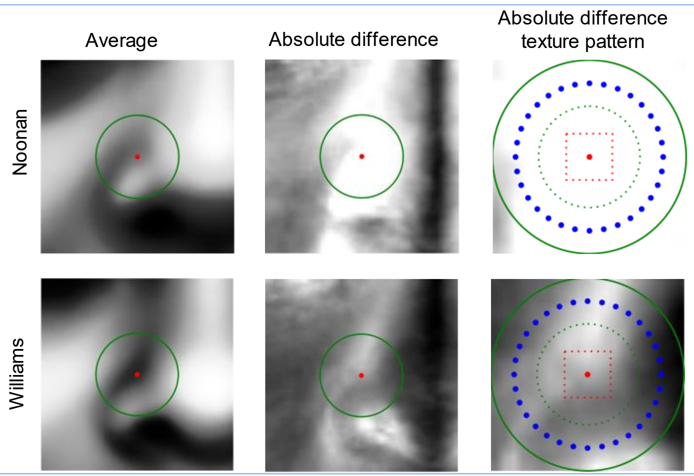
</td><td>4.42E-01</td></tr>
<tr><td colspan="3">Interpretation: Patients with Noonan syndrome present more asymmetric nasal alas than patients with Williams-Beuren syndrome.</td></tr>
<tr><td>Asymmetry of texture at lower eyelids (R1)</td><td>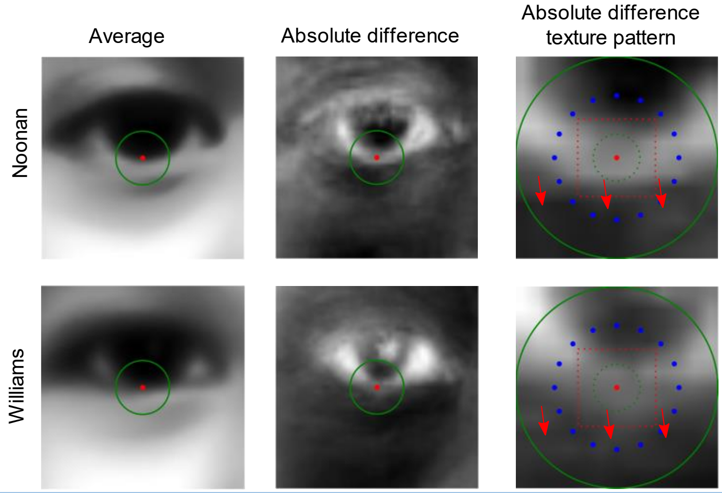
</td><td>4.49E-01</td></tr>
<tr><td colspan="3">Interpretation: Patients with Williams-Beuren syndrome have more asymmetric palpebral fissures than patients with Noonan syndrome.</td></tr>
<tr><td>Asymmetry of texture at oral commissures (R3)</td><td>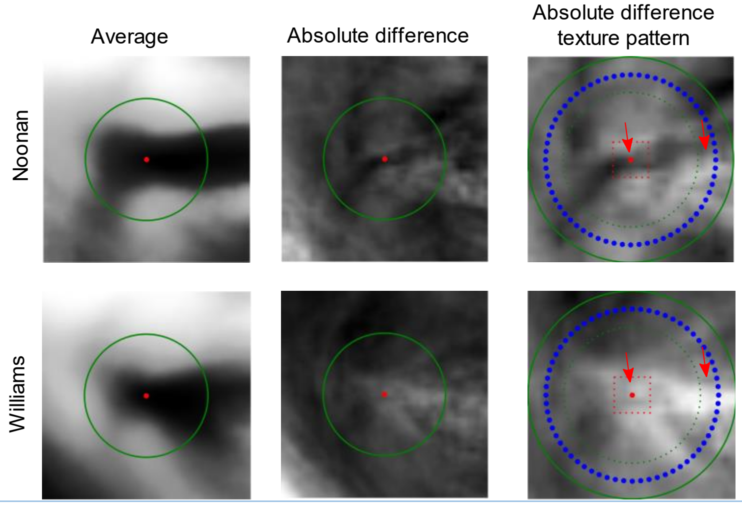
</td><td>5.96E-01</td></tr>
<tr><td colspan="3">Interpretation: Patients with Williams-Beuren syndrome present a more asymmetric mouth than patients with Noonan syndrome.</td></tr>
</table>

<table>
<tr>
<td>Asymmetry of texture at lateral of nasal bridge (R2)</td>
<td>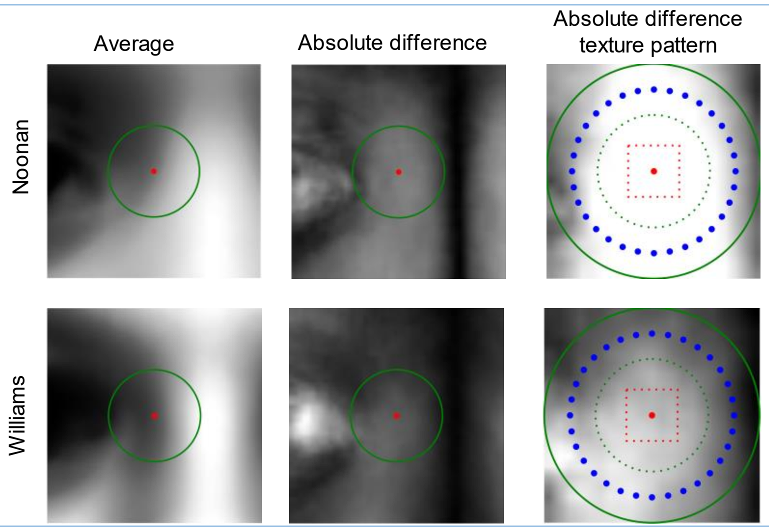
</td>
<td>6.62E-01</td>
</tr>
<tr>
<td colspan="3">Interpretation: Patients with Noonan syndrome present a more asymmetric nasal bridge than patients with Williams-Beuren syndrome.</td>
</tr>
</table>
